# Gender differences in teenager bullying dynamics and predictors of peer-to-peer intimidation

**DOI:** 10.1016/j.heliyon.2023.e20243

**Published:** 2023-09-16

**Authors:** Sergio A. Useche, Raquel Valle-Escolano, Eliseo Valle, Natura Colomer-Pérez

**Affiliations:** a University of Valencia, Spain; b Department of Constitutional Law, Political and Administrative Sciences, University of Valencia, Spain; c Department of Education and School Management, University of Valencia, Spain

**Keywords:** Educational policy, Gender perspective, Multi-group structural equation modeling, School bullying, Teenagers

## Abstract

Thanks to several previous efforts, school peer-to-peer bullying is nowadays considered a major issue for educational dynamics, research, and policy. Specifically in the field of research, bullying assessment tools have been gaining ground in recent years. Among them, the School Bullying Questionnaire (CIE-A) stands out. This is a teenager-targeted scale assessing bullying dynamics from a three-factor approach (i.e., victimization, symptomatology, and intimidation). However, to date, no previous study using similar tools has followed a gender perspective, and this shortcoming may hinder the effectiveness of policies and actions to face school bullying. The core aim of this study was to examine the effect of gender on teenagers' bullying-related factors and intimidation outcomes. This cross-sectional study analyzed the data provided by a gender-weighted sample of 770 Spanish teenagers with a mean age of *M =* 14.25 (*SD* = 1.53) years. They responded to a questionnaire that included the CIE-A together with other variables theoretically related to bullying dynamics, such as risk perception, sensation seeking, life satisfaction, and family conflict. Apart from typical inter-group comparisons, data were analyzed through a multi-group structural equation modeling (MGSEM) approach. Regarding bullying experiences, male teenagers have shown greater involvement in both victimization (passive bullying) and intimidation (active harassment) behaviors. On the other hand, females self-reported greater symptomatology in passive bullying scenarios, despite being less frequently involved in them. Further, the MGSEM showed good fit values (RMSEA <.08; all incremental coefficients >0.90) and theoretical plausibility, also depicting a set of structural mechanisms differentially explaining active peer-to-peer intimidation behaviors across genders. For instance, while risk perception is a significant predictor of intimidation only among females, sensation seeking plays a predictive role among male teenagers, but not for their female counterparts. The results of this study suggest that teenagers’ engagement in active bullying may be substantially different when approached from a gender perspective, finding key divergences in the variables that predict bullying-related behavioral outcomes. The outcomes of this research highlight the need to take into account gender differences, as well as extracurricular issues that influence intimidation dynamics, in potential bullying-related interventions.

## 1 Introduction

Apart from being an indisputable coexistence problem in school contexts around the world, bullying continues to be a public health problem with long-term effects for both direct and indirect victims, bullies, and the educational system itself [1,2]. Although in past decades bullying was approached more as an *individual issue*, socioecological theories have systematically shown the relevance of different social and contextual issues for its prevention and intervention [[3], [4], [5], [6], [7]].

Studies on the dynamics and prevalence of peer bullying suggest that it still occurs primarily in a face-to-face mode and can involve (amongst others) physical harm, verbal taunting, social exclusion, and relational aggression. In terms of prevalence, bullying behaviors typically affect between 2% and 16% of schooled teenagers, even though some studies report occasional victimization incidents involving up to 80% of them [[8], [9], [10]]. From a cross-national approach, a study conducted in 54 countries points out that bullying intimidation, victimization, and their consequences are still rising among adolescents, indicating a prevalence of serious injury ranging from 45.1% to 50.1% [11].

In addition, and even though some recent empirical studies suggest key differences, the evidence as a whole still lacks a gender-based perspective to address the dynamics and outcomes of bullying. As a figure, although it is estimated that the global prevalence of bullying among female adolescents ranges between 28.2% and 30.4%, it can increase to 30.5% and 34.8% in the case of their male counterparts [8,9]. Apart from the gender differences mentioned, some specific characteristics of bullying such as the exercise of physical, verbal, and symbolic violence have been mainly related to the case of peer-to-peer bullying among males, while females -acting more indirectly-tend to be more likely to suffer relational harassment expressions, such as being a victim of spread rumors or being excluded and/or ignored by others [12].

Regarding the short-term consequences of school bullying, the literature consistently describes physical, mental, and social health outcomes among bullies and their victims, as well as connections with negative psychological symptomatology and other health problems, e.g., attention-deficit hyperactivity disorder (ADHD), psychological distress, poor self-esteem, low life satisfaction, relational problems, eating disorders, anxiety, and depression [[13], [14], [15], [16], [17], [18], [19]].

In the mid- and long-term, school bullying also predicts increased risks of violence and abuse in adulthood [20], deliberated self-harm, and suicidal thoughts that sometimes materialize [[21], [22], [23], [24]]. All in all, although it is imperative to timely address the dynamics of bullying, several studies highlight the lack of empirical knowledge on its specific issues, such as gender differences and potentially unseen variables present in the micro and macro social systems of adolescents [1,25].

### 1.1 “Not all bullies/victims are the same”: the importance of targeting bullying patterns

When analyzing the interactions between contextual and individual issues of bullying from ecological theories, risk factors from different spheres such as family, personality, and social environment seem critical to address bullying from a holistic perspective [26,27]. For instance, only in recent years, typical teenage personality issues such as sensation seeking have been related to intimidation dynamics in bullying-related literature [28,29].

Parallelly, recent evidence suggests that bullying rates vary according to age and tend to peak in middle school, about 12–16 years [30], being more prevalent worldwide among early adolescents, especially if compared with late adolescents, according to previous cross-national studies [31]. For this reason, most research tools have focused on these ages to develop assessing methodologies for peer-to-peer intimidation, including the School Bullying Questionnaire (CIE-A) [32]. Regarding other age-based prevalence rates, a recent study [33] reported a prevalence (victimization and perpetration) among youth students that was approximately 50% lower than that observed among pre-adolescent students in Brazil. In Spain, the role of *bully* has shown a higher prevalence between 11 and 15 years of age (between 1st and 4th secondary grades) [34], coherently with the data reported by studies addressing other Hispanic samples [35,36].

Another pivotal factor affecting bullying victimization reported by the literature is the idiosyncrasies of the family environment and, specifically, the existence of a low level of parental education in social norms [37]. Furthermore, several studies have shown that being involved in peer victimization, either as a victim or perpetrator, is more prevalent in children and adolescents of lower socioeconomic status [38]. This entanglement could be inflicted in different ways, as parents facing this situation tend to become less involved in their children's education, practicing lower levels of academic socialization with them, along with less participation in social activities [39,40].

### 1.2 Gender seems to count a lot … but ‘not yet’ for educational policy?

In terms of gender, recent research [34] reported that there could be a higher frequency of female *non-victim* + *non-bully* profiles compared to males, while male teenagers are typically more frequently engaged in *bully* + *victim* roles than females.

Likewise, gender differences in bullying have been explored in different studies, among which some suggest that gender might be not statistically associated with the probability of suffering or being a perpetrator of traditional bullying, but rather with its dynamics [9,41]. In contrast, another study [42] suggested that there are male-female differences in traditional school bullying, in which gender seems to have a direct effect on specific bullying victimization, arguing that *Machiavellianism* and school climate may largely explain these differences. According to this study, boys are more likely to be involved in both perpetration and victimization of traditional school bullying.

At a theoretical level, it is known that schools that have developed a favorable pedagogical and social climate (usually involving gender-based actions) tend to have fewer peer-to-peer intimidation records [2]. In addition, different systematic reviews have concluded that school bullying prevention programs work better if key differential factors of teenagers (e.g., gender and extra-school social relationships) are addressed [43,44]. However, so far bullying remains a complex and considerably unexplored phenomenon in which several known and unknown factors might be involved, although the school context is expected to be a safe space for education, socialization, acceptance, and protection [45].

Finally, and regarding practical issues, it is worth mentioning that although the literature commonly endorses the idea that prevention programs at school must consider gender roles, norms, and frequently gendered communication patterns to propose gender-specific interventions that account for such differences, the development of applied experiences testing these assumptions (raised from different paradigms and theoretical approaches) remains pending in most geographical contexts, including Spain [[46], [47], [48]], thus contributing to perpetuating the gap in terms of both evidence and action, something necessary for making adequate decisions in educational policy.

### 1.3 Objectives and hypotheses

Bearing in mind the aforementioned considerations, the core aim of this study was to examine the effect of gender on teenagers’ school bullying-related factors. For this purpose, we followed the next three specific aims:

(i) To assess gender differences in a series of theoretically supported bullying-related factors and outcomes (see section 3.1).
(ii) To examine the multivariate relationships among the three core bullying dimensions of the CIE-A (see section 3.2).
(iii) To assess the structural differences in peer-to-peer intimidation behaviors from a gender-based perspective, considering a set of theoretically grounded demographic and psychosocial variables as their potential predictors (see section 3.3).

Regarding the hypotheses of the study, and taking into account the aforementioned previous research that supports the influence of the study variables on active bullying, we expected to find that:

(i) There are significant differences regarding three core bullying-related factors (i.e., victimization, symptomatology, and intimidation behaviors) between male (boys) and female (girls) teenagers. This non-directional hypothesis is supported by the fact that previous studies have concluded that bullying-related factors may have differential outcomes depending on teenagers' gender issues [9,42].
(ii) Literature-based demographic and psychosocial predictors of bullying would significantly explain peer-to-peer intimidation scores. This assumption is based on a series of commonly significant bullying predictors documented in the empirical literature, including sensation seeking, family conflict, and psychological health issues, such as distress, and life satisfaction [2,26,29,49,50].

However, (*iii*) these variables might have a differential influence on intimidation (active bullying) behaviors if gender is considered as an analytical category. In addition to differential propensity patterns related to aggression between genders, previous studies have suggested that factors such as risk perception, personality (especially sensation seeking), and health-related issues may differentially affect active bullying outcomes [42,43,[51], [52], [53]].

## 2 Methods and materials

### 2.1 Sample

For this cross-sectional study, the data were collected from a weighted sample of *n* = 770 Spanish teenagers: 385 (50%) participants identified themselves as boys (male teenagers), and 385 (50%) as girls (female teenagers). The detailed set of basic features of the study sample is available in Table 1. The sample had a mean age of *M =* 14.25 (*SD* = 1.53) years. The age range of the sample was 12–18 years and all students were currently enrolled in an academic course between the 1st of secondary – 2nd of high school.

**Table 1:** Descriptive demographic data of the study sample.

Variable	Group/value	n	%
**Gender**	Female (Girl)	385	50%
	Male (Boy)	385	50%
**Current grade/school year**	1st Secondary	196	25.5%
	2nd Secondary	206	26.8%
	3rd Secondary	88	11.5%
	4th Secondary	171	22.2%
	1st High School	102	13,20%
	2nd High School	5	.6%
**Do you own a smartphone?**	Yes, with free/unattended use	504	65.5%
	Yes, but with restricted/supervised use	236	30.6%
	No	30	3.9%
**Use of social networks**	Never	35	4.5%
	Rarely	27	3.5%
	Weekly or less	28	3.7%
	Several times a week	67	8.7%
	Once a day	76	9.9%
	Several times a day	537	69.7%

Concerning technological trends, it stands out that 96.1% of participants (teenage students) already owned a smartphone. Among them, about one-third stated its use was somewhat restricted (e.g., limited functionality by an app) or supervised by parents (e.g., only allowed to use it at home). On the other hand, approximately two-thirds of mobile phone owners stated a relatively free right to use their devices. Approximately 70% of participants declared a greatly frequent social network use, while only about 10% accessed them less than once a week (including teenagers not using them at all).

### 2.2 Study design, setting, and procedure

This cross-sectional study, conducted in Spain, analyzed the data retrieved from an extensive sample of secondary and high school students. These data were collected during the year 2020. As the target population of this study is essentially underaged (secondary level students are rarely older than 18), the research setting implied the first step of gathering written (i.e., informed consent) permissions from relevant stakeholders (parents’ associations, school teachers, and program coordinators). Having counted on a broad research staff, the data collection procedure was carried out during approximately two months (not including vacation nor exam seasons) of one single academic year, to avoid essential confounding effects and dual partaking. The questionnaires were prepared on paper, and a single format (written in the official language) was used uniformly in all the participating centers.

Regarding data protection issues, only generic demographics useful to characterize participants (e.g., age, gender, educational year) were gathered, and no personal information potentially allowing us to identify them was considered. Further, data processing followed a group-based and never participant-centered perspective. Before starting the completion of the research form, and to prevent potentially biased responses, we emphasized: (*i*) the anonymity of the form; (*ii*) the scientific character of the study; (*iii*) the importance of answering honestly to all questions, (*iv*) the non-existence of right or wrong answers; as well as (*v*) the possibility of asking for clarification/help from a research associate, if necessary. The development of the data collection phase passed without substantial difficulties and the dropout rate of participants once the completion of the questionnaire had begun was less than 1%.

As for preliminary statistical power and technical checks, an a priori power analysis showed an initial minimum sample size of approximately n = 380 individuals, assuming a level of confidence of 95%, a maximum margin of error of 5% (α = 0.050), and a beta (β) of 0.200, which allows for 80% power. However, specialized literature advises seeking greater sample sizes if the sample will be split into two or more groups, or if non-probability samples are used, even if they are homogeneous [54,55]. Therefore, and considering the collective application of the form, the support given by teachers, and the high willingness to partake in the study, the response rate was almost 90% of the invited students, reaching a final number of 770 fully completed questionnaires.

### Ethical approval

The research protocol of this study was examined and approved by a specialized Ethics Committee, granting its compliance with the Declaration of Helsinki, as well as with the ethical considerations needed to approach the target population (Ethics Committee of the 10.13039/501100003508University of Valencia, IRB H01535548125595).

### 2.4 Study questionnaire

The questionnaire used for this research was administered in Spanish (native language) and consisted of four sections:

The first section aimed at gathering individual and demographic information, including students' age, gender (understood in this study as the self-declared identity of our partakers, which may or may not correspond to the person's sex at birth; female/male/non-binary), current school grade, general academic average [0–10] of the last school year approved (used as an academic performance indicator), and technology-related questions, including owing/not owning a mobile phone, and their frequency of connected features and use of social media, self-rated on a [0–5] scale.

School bullying-related factors were assessed through the School Bullying Questionnaire (CIE-A) [32], and validated in Ref. [48]. The CIE-A is a frequency-based Likert scale composed of 36 questions, using a 3-point scale, ranging from 0 (never) to 10 (very often). It measures three factors: F1 (*Victimization*, understood as common bullying situations suffered by the respondent; *α* = 0.859) and F2 (*Symptomatology*, consisting of common psychological and behavioral signs or reactions to bullying situations; *α* = 0.870) related to passive bullying experiences, while F3 (*Intimidation*, understood as the performance of harassing behaviors towards peers; *α* = 0.902) measures individuals’ involvement in active bullying experiences.

In the third part of the questionnaire, we inquired about individual factors. Namely, Sensation seeking was measured through the Stephenson's Brief Sensation Seeking Scale (BSSS) [56], an 8-item Likert questionnaire (*α* = 0.880) assessing young and adult individuals' need for novel and intense experiences (i.e., sensation-seeking personality trait), following a 0 (strongly disagree) to 4 (strongly agree) measurement scale. Risk perception was measured through the General Risk Perception Subscale (GRPS) [48], which is a 7-item Likert form (*α* = 0.853) in which the degree of risk perceived in objective risk factors is assessed, from 0 (lower risk perception) to 4 (higher risk perception).

In the fourth section, parents' conflict was measured through the Interparental Conflict Inventory (ICI) [57]. This is a 10-item Likert questionnaire (*α* = 0.821) assessing teenagers' perceptions of conflict in their parents' daily interaction. General psychological health was measured through Goldberg's General Health Questionnaire (GHQ) [58]. The 12-item version of this Likert scale (α = 0.849), endorsed for use among adolescents provides an overall psychological distress measure. Finally, Life Satisfaction was measured through Diener's Satisfaction with Life Scale (SWLS) [59], a Likert form (α = 0.807) composed of 5 items assessing individuals' global life satisfaction as a single factor based on a 7-point scale.

### 2.5 Statistical analysis strategy (data processing)

After a careful curation of the data, all the basic descriptive analyses were conducted. This allowed us to get the dimensional scores and descriptive statistics (i.e., mean, standard deviations, standardized errors) of the scales. Once the basic parameters were tested, comparative tests (Welch) were carried out to compare gender differences on bullying-related factors. Welch's tests are Student's t-based robust procedures entailing several advantages over ANOVAs for self-report questionnaire outcomes, particularly in non-normal distributions, or among groups whose variances are not necessarily equal.

As for structural analyses, the MGSEM (Multi-Group Structural Equation Modeling) procedure was used to assess the multivariate relationships between (on the one hand) teenagers’ demographic and psychosocial factors, e.g., risk perception, sensation seeking, family issues, life satisfaction –used as independent variables or *predictors*– and (on the other) their peer-to-peer-intimidation patterns, used as a dependent or *predicted* variable–. From a statistical point of view, this literature-based technique is more accurate than discretely assessing gender values as unconnected populations, given that it considers the full sample parameters for fitting the models, drawing covariances between exogenous factor errors, and controlling by third variables (in this case age and school year), thus reducing the potential impact of demographic confounders on the model's outcomes.

Further, although betas (*β*) are standardized coefficients and can be assumed as controlling for the effects of other predictors within the model, the error terms of highly correlated exogenous variables were covaried, making them able to account for their systematic associations without implying non-literature-endorsed causal relationships. The model significance criteria were differentially set at: *p* < .05 (*); *p* < .010 (**); and *p* < .001 (***).

Apart from representing the theoretical assumptions of the model in a theoretically suitable way, the goodness-of-fit was evaluated through a broad set of literature-supported indices from different families [60,61]: χ^2^/degrees of freedom, minimum discrepancy ratio (CMIN/df); Normed Fit Index (NFI); Confirmatory Fit Index (CFI), Tucker-Lewis Index (TLI), Incremental Fit Index (IFI) and Root Mean Square Error of Approximation (RMSEA). The quantitative cut-off criteria followed were coherent with those used the most in specialized literature: CMIN/df ratio <5.000; ordinal/incremental coefficients (NFI/CFI/TLI/IFI) > 0.900; and RMSEA <0.080 [62].

All descriptive statistical analyses were conducted on IBM SPSS (Statistical Package for Social Sciences; version 28.0). Graphical analyses were conducted in Sigma Plot (version 13.0), and multi-group structural equation modeling was performed through IBM SPSS AMOS (version 28.0).

## 3 Results

### 3.1 Descriptive outcomes

The descriptive statistics of the study variables are presented in the mid-columns of Table 2. Further information on the scales used for measuring each variable is available in section “*2.3 Study questionnaire*”. Overall, robust comparative analyses yielded several significant differences between female and male teenagers. While risk perception and sensation seeking did not show significant gender-based differences (and therefore had the lowest both η^2^ and ω^2^ effect sizes) all the other variables assessed in the research procedure have shown significantly differential values (see right columns of Table 2).

**Table 2:** Descriptive statistics and robust mean comparisons between genders.

Variable	Group	M	SD	Statistic^a^	df1/df2^b^	η^2^	ω^2^	Sig.
Risk Perception	Female	3.35	1.01	2.206	1/749040	.003	.002	.138
	Male	3.24	1.18					
Academic Performance	Female	7.72	1.93	13.382	1/741442	.017	.016	***
	Male	7.16	2.23					
Sensation Seeking (SS)	Female	3.70	1.27	1.588	1/767755	.002	.001	.208
	Male	3.81	1.25					
ICT Interaction	Female	4.57	1.11	43.358	1/660984	.053	.052	***
	Male	3.93	1.05					
Parents' Conflict	Female	2.70	1.36	5.563	1/740109	.007	.006	*
	Male	2.95	1.66					
Psychological Distress	Female	22.06	5.34	42.094	1/751086	.052	.051	***
	Male	19.73	4.59					
Life Satisfaction	Female	21.75	5.20	4.434	1/736695	.006	.005	*
	Male	22.51	4.64					
F1: Victimization	Female	2.38	3.27	24.085	1/675422	.033	.031	***
	Male	3.75	4.18					
F2: Symptomatology	Female	5.48	3.81	17.658	1/667.231	.024	.023	***
	Male	4.01	5.22					
F3: Intimidation	Female	.53	3.40	39.261	1/526369	.053	.051	***
	Male	1.80	2.76					

*Notes:*^a^Asymptotically F distributed; ^b^df = Degrees of Freedom; *The difference is significant at the level <0.050; η2 = Eta-squared coefficient; ω2 = Omega-squared fixed-effect; ***The difference is significant at the level <0.001.

Namely, female teenagers self-report higher average scores for academic performance (W_*t*_ = 13.382; *p* < .001) and a greater ICT involvement frequency (W_*t*_ = 43.358; *p* < .001). Self-reported parent conflict had higher scores among males (W_*t*_ = 5.563; *p* < .050). In terms of mental health-related indicators, females also show slightly (but significantly) higher scores for psychological distress (W_*t*_ = 39.261; *p* < .001) and a lower mean of life satisfaction (W_*t*_ = 4.434; *p* < .050) when compared with males.

**Fig. 1:**
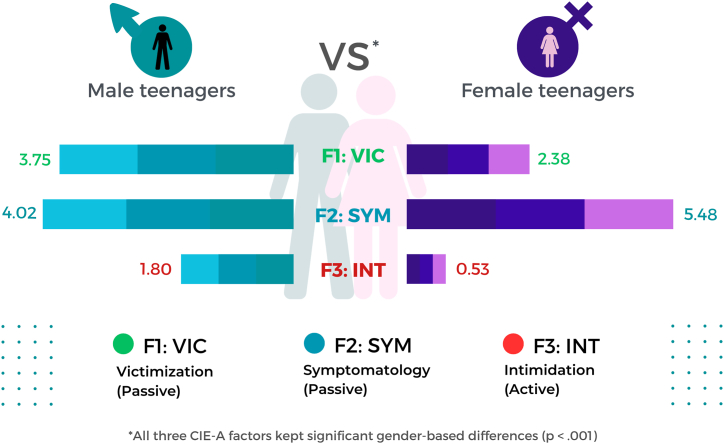
Gender-based differences in bullying-related (CIE-A) factors (mean values).

Also, the three bullying-related CIE-A factors addressed show gender differences. On the one hand, male teenagers tended to self-report greater bullying victimization (W_*t*_ = 24.085; *p* < .001) and intimidation (W_*t*_ = 39.261; *p* < .001) rates than their female counterparts. On the other hand, females were those scoring the highest in symptomatology (bullying affectation; W_*t*_ = 42.094; *p* < .001). The differences gathered regarding these three variables are graphically shown in Fig. 1.

### 3.2 Relationships among bullying-related factors

**Fig. 2:**
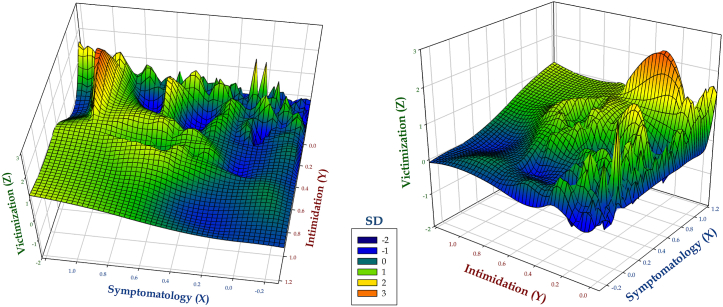
Tridimensional (XYZ) associations of CIE-A bullying-related factors. Note: The scoring scale has been transformed to standardized scores (SDs), in order to favor fair comparability among the three variables included in the analysis.

Specifically addressing the second aim of this study, there were assessed the multivariate links among the three peer-to-peer bullying-related factors measured by the School Bullying Questionnaire. This is similar to a graphical clustering procedure, entailing the advantage of visually addressing the relevant (associative, although not predictive) intersections among the three factors analyzed, whose critical locations are labeled with a red-like color, similar to conventional heat plots. The core outcomes of such analysis are shown in Fig. 2.

Overall, the XZ (symptomatology*victimization) intersection shows that high-victimization scenarios (F1) are associated with a substantial increase of symptomatology (F2; quadrants X[0.8–1.0], Z[1.0–2.2]), whose high slope (measuring the rate of change in a variable as other variable changes) suggests a considerable concentration of cases meeting greater values in both cases. Also, both the ZY (victimization*intimidation) and XY (symptomatology*intimidation) intersections depict the low frequency of cases in which passive bullying factors (F1 and F2) are actually associated with the active exercise of bullying.

### 3.3 Multi-group structural equation modeling

Based on the aforementioned theoretically-based assumptions (please see *Introduction*), the effect of gender on self-reported active bullying behaviors, i.e., intimidation behaviors (F3), was examined using a multi-group structural equation modeling (MGSEM) approach. In addition to being substantially different from comprising a “dummy” category (e.g., “being a male”) as a discrete variable within a structural equation model, multi-group analysis accounts for the effect of the exogenous factors on the dependent variable for each group.

**Fig. 3:**
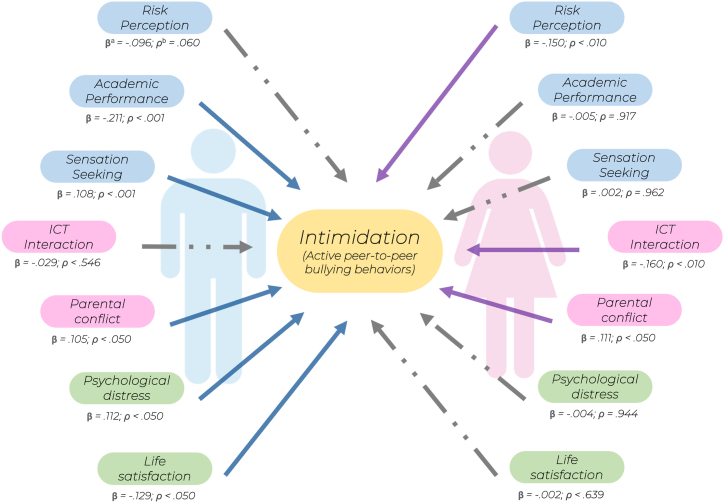
Two-group structural model showing standardized pathway coefficients for peer-to-peer intimidation behavior: male teenagers (left) and female teenagers (right). *Notes:*^a^Standardized (Beta) estimates; ^b^Test p-values (paths with coefficients below .050 can be interpreted as statistically significant with a 95% confidence level). Discontinuous arrows indicate non-significant paths. Additional data on the model pathways and coefficients are presented in Table 3.

In this sense, the aggregated sample was split into two gender-based (50-50) groups (reference categories), both of them with a suitable sample size for the intended analysis and a clear proportionality for the comparative exploration. Also, in order to avoid basic demographic bias, the model controlled for age and school year. The resulting Structural Equation Model, simultaneously fitted for both gender groups or categories (*x*_2(24)_ = 27.323, *p* = .290; CMIN/df = 1.138; NFI = 0.940; CFI = 0.992; TLI = 0.980; IFI = 0.992; RMSEA = 0.013, IC 95% [0.001-0.025]), is graphically presented in Fig. 3.

Regarding MGSEM core outcomes, the effect of exogenous variables on peer-to-peer intimidation behaviors showed differential trends between male and female teenagers. The *β* standardized path coefficients of the model (see Table 3 and values next significant paths in Fig. 3) suggest key gender-based structural differences.

**Table 3:** Multi-group SEM Model to predict self-reported intimidation (active bullying) behaviors.

Group 1: Males						
Path			SPC	S.E.	C.R.	P
Intimidation	⟵	Risk Perception	−.096	.009	−1.879	**
Intimidation	⟵	Academic Performance	−.211	.008	−4.081	***
Intimidation	⟵	Sensation Seeking	.108	.008	2.273	*
Intimidation	⟵	Interaction with ICTs	−.029	.006	−.604	.546
Intimidation	⟵	Parental Conflict	.105	.003	2.029	*
Intimidation	⟵	Psychological Distress	.112	.014	2.094	*
Intimidation	⟵	Life Satisfaction	−.129	.012	−2.411	*
**Group 2: Females**						
**Path**			**SPC**^**1**^	**S.E.**^**2**^	**C.R.**^**3**^	**p**^**4**^
Intimidation	⟵	Risk Perception	−.150	.005	−2.977	**
Intimidation	⟵	Academic Performance	−.005	.004	−.104	.917
Intimidation	⟵	Sensation Seeking	.002	.004	.048	.962
Intimidation	⟵	Interaction with ICTs	−.160	.004	−3.02	**
Intimidation	⟵	Parental Conflict	.111	.002	2.172	*
Intimidation	⟵	Psychological Distress	−.004	.005	−.071	.944
Intimidation	⟵	Life Satisfaction	−.025	.005	−.470	.639

*Notes:*^1^ SPC = Standardized Path Coefficients (can be interpreted as β-linear regression weights); ^2^ S E. = Standard Error; ^3^ C R. = Critical Ratio; ^4^ p-values; *Significant at the level <0.050; **Significant at the level <0.010; ***Significant at the level <0.001.

In the case of males, neither risk perception nor ICT interaction scores significantly predicted intimidation scores. Rather, there were significant effects from academic performance (*β* = −0.211; *p* < .001); parents’ conflict (*β* = 0.105; *p* < .050); sensation seeking (*β* = 0.108; *p* < .001); and both mental health indicators: psychological distress (*β* = 0.112; *p* < .050) and life satisfaction (*β* = −0.129; *p* < .050). Said otherwise, intimidation likelihood was better predicted in scenarios characterized by lower academic performance and life satisfaction, in addition to greater parental conflict and psychological distress.

In the case of female teenagers, and compared to the males' model structure, the exogenous variables having a significant association with active peer-to-peer bullying behaviors substantially differ, although few commonalities were kept. Specifically, neither academic performance, sensation seeking, nor mental health indices (i.e., psychological distress and/or life satisfaction) has significant effects on the endogenous variable. Nevertheless, females’ risk perception (*β* = −0.150; *p* < .010), as well as their ICT interaction levels (*β* = −0.160; *p* < .001), were differential significant predictors. Further, although in common with male teenagers, the presence and degree of parental conflict significantly and positively predicted peer-to-peer intimidation.

## 4 Discussion

Based on the data provided by 770 Spanish participants, this paper examined the effect of gender on teenagers’ school bullying-related factors. Overall, and aside from a set of interesting descriptive differences, this study used (for the first time) an MGSEM approach to differentiate active predictors of peer-to-peer bullying based on gender.

Initially, the descriptive gender-based differences found in this study suggest a considerably *gendered* state of affairs regarding teenagers’ self-reported perception of bullying victimization and symptomatology (passive factors) and intimidation (active bullying behaviors). The outcomes of these comparisons, carried out using robust mean tests, show great agreement with the findings of both qualitative [25] and quantitative [34,52] previous studies, regarding the greater involvement in active bullying behaviors among male students, compared with their female counterparts. Coherently, a recent study [63] has found a greater affectation among females as a consequence of their exposure to relational peer-to-peer bullying behaviors in a passive role.

Further, the multi-group structural equation modeling (MGSEM) was useful in finding empirical evidence that supports the hypothesis that there are key differences related to gender in the explanation of bullying dynamics, finding how a set of literature-based variables may differ influencing their self-reported engagement in intimidation behaviors, as will be summarized in the following paragraphs of this paper.

### 4.1 Summary: gender-based structural differences and similarities in peer-to-peer intimidation behaviors

The core splitting factor addressed in this study was gender. In this regard, our research aimed to study the structural differences (and similarities) between male and female teenagers in various factors, which theoretically influence their participation in bullying behaviors. The MGSEM model allowed us to determine, specifically for each literature-based exogenous variable, that:

- *Risk perception.* This first variable appended in the graphical model had a differential effect on intimidation outcomes between female and male teenagers. Specifically, the predictive path was significant for females but non-significant for their counterparts. From a literature point of view, and apart from advancing that risk perception is a common issue in the explanation of risk-taking behaviors (including school bullying), some studies have previously argued that, regardless of risk awareness, male adolescents tend to assume more risks and be less sensitive to negative outcomes than females [64]. In other words, and also given social roles and attitudes formed in society, males' behavioral potential could be less inhibited regardless of still perceiving risky behavior outcomes [25,42].
- *Academic performance.* Although the literature from a few decades ago assumed that the likelihood of engaging in bullying behaviors was closely linked to poor school performance, subsequent studies have provided evidence that this is now quite relative. For example, studies such as [34,65] show that, although academic performance tends to be lower among *bullies* (as compared to the *bullied*), gender, age, and other third factors could better explain these trends. Accordingly, we have found in this study that (poor) academic performance exerts a significant effect on intimidating behaviors in the case of male bully profiles, albeit not in the case of females.
- *Sensation seeking.* Similar to academic performance, sensation seeking has a significant effect on active bullying behavior among teenage boys, but not among girls. In particular, and unlike the high-order ones (whose study is advisable rather from young adulthood), this personality trait has been remarked as a major issue for adolescent behavior throughout the previous literature. In this regard, there are some applied studies on bullying [28,29] endorsing the assumption that *high-sensation seeking* adolescents (but especially males) get more frequently engaged in active bullying dynamics, as well as in other harmful behaviors, including substance use [66], cybernetic bullying [67], and extra-school aggression [29,50].
- *ICT interaction.* While in many fields increased interaction with information and communication technology (ICTs) is commonly associated with negative outcomes [68,69], in some others –and despite their evident risks– individuals' access to information, social networks, and socially open scenarios during the *information age* have been related to certain positive and risk-inhibiting outcomes [70,71]. In the case of this study, the results suggest that this would be especially beneficial among female teenagers, although this same statistical pathway remains non-significant for males. A potential literature-based explanation for this difference could be, according to studies such as [72,73], the dissimilar use (in terms of motives, purposes, and dynamics) of such “connected scenarios” among genders, whose support-seeking role might be observed more frequently among female teenagers [74].
- *Parental conflict.* A key commonality of this set of structural comparisons is that the presence and perceived degree of parental conflict affects engagement in bullying behaviors, in both male and female teenagers. Far from being an unexpected result, this particular outcome has been previously supported by studies addressing the predictors of violent and defiant behaviors among young individuals [50,75], the frequency, intensity, and consequences of which are consistently associated with violence and conflict in the microsocial system of adolescents [1,37].
- *Mental health indicators.* As for the last two variables included in the model, a “worse” mental health status (e.g., higher rate of psychological distress and lower life satisfaction) has been shown to influence engagement in intimidation behaviors, even though these two paths were statistically significant only in the case of males. Accordingly, previous studies have suggested that, apart from key gender differences, mental health issues may be problematically *gendered*, as male adolescents commonly suffer from but under-report these complaints as a consequence of social issues, stigmas, and predefined roles [49,76]. In addition, most sources dealing with this relationship have stressed the need to promote timely and effective interventions to prevent typical harmful behaviors resulting from teenagers' mental health issues, such as self-harm, aggression, and peer-to-peer intimidation [74,77].

## 5 Conclusions

This study aimed to assess bullying-related dynamics and the predictive effect of a series of theory-based variables on peer-to-peer intimidation from a gender perspective. Its core conclusions are presented below, per the study aims:

Firstly, and regarding gender differences in dimensional scores, male teenagers have shown both greater victimization (passive bullying) and intimidation (active intimidation behaviors). However, female teenagers were those self-reporting greater symptomatology as a consequence of bullying exposure.

Secondly, the multivariate analysis of the CIE-A bullying factors suggests a low frequency of “passive + active” peer-to-peer bullying profiles (i.e., individuals playing both perpetrator and victim roles). Indeed, and although not in all cases, there is a frequent concordance between “passive” and “active” teenager bullying profiles.

Thirdly, the structural mechanisms explaining active peer-to-peer intimidation behaviors have shown several differences across male and female teenagers. Said otherwise, the MGSEM analyses show that teenagers’ engagement in active bullying dynamics can be substantially different if it is approached from a gender-based perspective.

### 5.1 Implications for policy

The outcomes of this research allow us to imply two policy implications to prevent, mediate, and alleviate teenagers’ peer-to-peer school bullying. On the one hand, gender differences seem to count on the explanation of its related dynamics, and therefore, they should be accounted for in bullying-related interventions. On the other, some of the common factors (e.g., parental conflicts) seem to exceed the school context, making it relevant to raise multi-level potential interventions.

### 5.2 Limitations of the study and further research

Although this research used a considerably extensive and balanced sample, robust comparative analyses were used, and both technical and theoretical assumptions were satisfactorily achieved, there is a set of potential limitations and bias sources that must be acknowledged.

Firstly, even though the 100% literacy of the target population, and the facts that (*i*) the study questionnaire was anonymous, and (*ii*) the non-existence of right or wrong answers was emphasized by our research staff, previous studies emphasized the potential prevalence of common method variance (CMV) as a typical result of using self-report methods for behavioral research [78]. This should be considered in interpreting the study results.

Secondly, it is known that bullying remains a very sensitive topic, and cultural issues may bias its perception, appraisal, and self-reporting patterns based on variables such as gender. Therefore, these cultural gender inequalities should be taken into account as both potential influencing and differentiating factors for school-based dynamics [31,41].

Thirdly, it is important to mention that previous research has consistently highlighted “order effects” (e.g., the influence of the order of the topics presented, or previously answered items on further responses) as a common constraint potentially influencing the outcomes of questionnaire-based research [79,80]. While in this study we followed common suggestions from literature to increase understandability, such as consistently ordering response options in ascending manner (i.e., going from the lowest (left) to the greatest (right) degrees of *agreement*, *frequency*, or *perceived risk*) and addressing generic information at the beginning of the questionnaire and most *sensitive* issues (e.g., self-rated health, life satisfaction) rather at its end, other potential order-related confusion factors can be listed.

For instance, issues such as school dynamics and personality matters (adolescence is known to be a critical stage for personality development, still requiring further crystallization) [26,29,66], addressed during the intermediate part of the form could also affect the response style of our partakers.

Further, the questionnaire was applied in a single standardized version, which resulted from its pilot application. Although it guarantees a uniform presentation of contents, some studies alternatively suggest using multiple randomized survey forms in both qualitative and quantitative research as a manner to reduce potential bias. Therefore, the outcomes of this study must be interpreted in consideration of these matters [80,81].

Finally, and as for further studies, the authors would like to underline two core actions aimed at improving related research processes and their outcomes:

(i) To pursue the statistical representation of *non-traditional* (constructed) genders, despite the sometimes low accessibility and sample size-related methodological challenges it may pose to researchers. This would be beneficial to enhance a greater understanding of the current bullying dynamics and scenarios, especially since recent studies have highlighted the great vulnerability of non-binary students to school harassment [9,82].
(ii) To increase the number and deepness of the insights potentially retrievable on this interesting issue through (e.g.) in-depth interviews, focus groups, and the use of mixed research methods. This might help to enhance a complementary understanding of gender-based differences in bullying dynamics.

## Author contribution statement

Sergio A. Useche: Conceived and designed the experiments; Performed the experiments; Analyzed and interpreted the data; Wrote the paper.

Raquel Valle-Escolano; Eliseo Valle, Ph.D: Performed the experiments; Contributed reagents, materials, analysis tools or data; Wrote the paper.

Natura Colomer-Pérez: Conceived and designed the experiments; Analyzed and interpreted the data; Wrote the paper.

## Data availability statement

Data will be made available on request.

## Declaration of competing interest

The authors declare the following financial interests/personal relationships which may be considered as potential competing interests: Sergio A. Useche reports a relationship with Elsevier that includes: board membership. The corresponding author (Dr. Sergio A. Useche), is an Associate Editor of the journal. This does not alter our adherence to the ethical policies of Heliyon. Further, the authors of this study declare the inexistence of competing interests.
